# Comprehensive characterization of central BCL-2 family members in aberrant eosinophils and their impact on therapeutic strategies

**DOI:** 10.1007/s00432-021-03827-9

**Published:** 2021-10-15

**Authors:** Timo O. Odinius, Lars Buschhorn, Celina Wagner, Richard T. Hauch, Veronika Dill, Marta Dechant, Michele C. Buck, Khalid Shoumariyeh, Philipp Moog, Juliana Schwaab, Andreas Reiter, Knut Brockow, Katharina Götze, Florian Bassermann, Ulrike Höckendorf, Caterina Branca, Philipp J. Jost, Stefanie Jilg

**Affiliations:** 1 Clinic and Policlinic for Internal Medicine III, School of Medicine, Technical University of Munich, Munich, Germany; 2 Centre for Translational Cancer Research (TranslaTUM), School of Medicine, Technical University of Munich, Munich, Germany; 3 Department of Medicine I, Faculty of Medicine, Medical Center, University of Freiburg, Freiburg Im Breisgau, Germany; 4 German Cancer Consortium (DKTK) Partner Site Freiburg, Freiburg im Breisgau, Germany; 5 Department of Nephrology, Clinic and Policlinic for Internal Medicine II, School of Medicine, Technical University of Munich, Munich, Germany; 6 Department of Hematology and Oncology, University Hospital Mannheim, Heidelberg University, Mannheim, Germany; 7 Department of Dermatology and Allergy, School of Medicine, Technical University of Munich, Munich, Germany; 8 Division of Clinical Oncology, Department of Medicine, Medical University of Graz, Graz, Austria

**Keywords:** Hypereosinophilia, Hypereosinophilic syndrome, CEL-NOS, EGPA, BH3-mimetics, Apoptosis, Venetoclax, MCL1, S63845

## Abstract

**Purpose:**

Hypereosinophilia represents a heterogenous group of severe medical conditions characterized by elevated numbers of eosinophil granulocytes in peripheral blood, bone marrow or tissue. Treatment options for hypereosinophilia remain limited despite recent approaches including IL-5-targeted monoclonal antibodies and tyrosine kinase inhibitors.

**Methods:**

To understand aberrant survival patterns and options for pharmacologic intervention, we characterized BCL-2-regulated apoptosis signaling by testing for BCL-2 family expression levels as well as pharmacologic inhibition using primary patient samples from diverse subtypes of hypereosinophilia (hypereosinophilic syndrome *n* = 18, chronic eosinophilic leukemia not otherwise specified *n* = 9, lymphocyte-variant hypereosinophilia *n* = 2, myeloproliferative neoplasm with eosinophilia *n* = 2, eosinophilic granulomatosis with polyangiitis *n* = 11, reactive eosinophilia *n* = 3).

**Results:**

Contrary to published literature, we found no difference in the levels of the lncRNA *Morrbid* and its target *BIM*. Yet, we identified a near complete loss of expression of pro-apoptotic *PUMA* as well as a reduction in anti-apoptotic *BCL-2*. Accordingly, *BCL-2* inhibition using venetoclax failed to achieve cell death induction in eosinophil granulocytes and bone marrow mononuclear cells from patients with hypereosinophilia. In contrast, *MCL1* inhibition using S63845 specifically decreased the viability of bone marrow progenitor cells in patients with hypereosinophilia. In patients diagnosed with Chronic Eosinophilic Leukemia (CEL-NOS) or Myeloid and Lymphatic Neoplasia with hypereosinophilia (MLN-Eo) repression of survival was specifically powerful.

**Conclusion:**

Our study shows that *MCL1* inhibition might be a promising therapeutic option for hypereosinophilia patients specifically for CEL-NOS and MLN-Eo.

**Supplementary Information:**

The online version contains supplementary material available at 10.1007/s00432-021-03827-9.

## Introduction

Eosinophil granulocytes play a pivotal role in multiple hematologic, rheumatic, and allergic diseases (O'Sullivan and Bochner 2018; Simon et al. 2020). After exclusion of reactive changes to the blood count such as seen in parasitic diseases, chronic primary eosinophilia is characterized by permanent eosinophil counts > 500/µl. Severe hypereosinophilia is defined as an absolute eosinophil count > 1,5 × 10^3^/µl. Analysis of the bone marrow including cytogenetic and molecular genetic results is needed to characterize the specific subtypes including chronic eosinophilic leukaemia not otherwise specified (CEL-NOS), myeloid and lymphatic neoplasia with hypereosinophilia (MLN-Eo), idiopathic hypereosinophilia (HE) and hypereosinophilic syndrome (HES; Shomali and Gotlib 2019). eosinophilic granulomatosis with polyangiitis (EGPA) represents a possible differential diagnosis even in patients with ANCA (*anti-neutrophilic cytoplasmic antibody*) negativity. Specifically in patients presenting with elevated peripheral blood (PB) eosinophil granulocytes, clinical manifestations of hypereosinophilia range from sole laboratory aberrations to manifest organ infiltration in any tissue or the bone marrow. Therapeutic options are often limited to symptomatic therapy and cytoreduction, mostly using corticosteroids. Clinical phase-III-trials show promising results in targeting interleukin-5 (IL-5; mepolizumab) or the IL-5 receptor α (benralizumab) within the scope of steroid-sparing eosinophil reduction (Kuang et al. 2019; Rothenberg et al. 2008; Wechsler et al. 2017). For MLN-Eo with fusion transcripts of *PDGFRA* and *PDGFRB*, tyrosine kinase inhibition (TKI) with imatinib is an approved therapeutic concept (Shomali and Gotlib 2019).

Deregulated cell death is a critical finding in patient with hypereosinophilia (Litvinova et al. 2008). Evading apoptosis is one of the hallmarks of cancer (Hanahan and Weinberg 2011) and targeting cell death resistance is a promising therapeutic strategy (Jilg et al. 2016). In a physiological setting, intrinsic or mitochondrial apoptosis is tightly controlled by the interaction between pro- and anti-apoptotic members of the BCL-2 protein family (Youle and Strasser 2008). Pro-apoptotic BH_3_-only proteins such as *BIM*, *PUMA* and *BID* can directly or indirectly activate the pore-forming pro-apoptotic BCL-2 family members *BAX* or *BAK* at the outer mitochondrial membrane to facilitate caspase activation (Willis et al. 2007). This process is inhibited by the anti-apoptotic BCL-2 family members such *as BCL-2*, *BCL-XL* or *MCL1* (Youle and Strasser 2008). In a number of hematologic neoplasms aberrations in BCL-2 family proteins is well known (Fernández-Marrero et al. 2016; Merino et al. 2018). This pathologic disbalance between anti-apoptotic and pro-apoptotic BCL-2 family proteins can be molecularly targeted with pro-apoptotic BH_3_-mimetics. Among these, venetoclax (ABT-199) has already been FDA approved for treatment of chronic lymphatic leukemia (CLL) and acute myeloid leukemia (AML). In addition, recently new molecules for selective inhibition of *MCL1* or *BCL-xL* are being tested for diverse disease entities (Bessou et al. 2020; Kotschy et al. 2016; Munkhbaatar et al. 2020).

Despite extensive interdisciplinary literature search no data set was found comparing the BCL-2 proteins expression pattern of aberrant eosinophils and healthy controls in a larger approach and head-to-head. Recently published data in a high-impact journal e.g. mainly focused on the impact of pro-apoptotic *BIM* only (Kotzin et al. 2016). Using RT-qPCR, we therefore first quantified RNA levels of BCL-2-family members to evaluate differences between normal and “aberrant” eosinophils. Second, we analyzed the effect of the BH_3_-mimetics ABT-199, ABT-737, WEHI-539 and S63845 on eosinophil granulocytes from healthy donors compared to an enlarged cohort of patients with hypereosinophilic disorders. Third, we focused on the therapeutic impact of BCL-2 protein inhibition on myeloid progenitor cells extracted from the BM and cultivated ex vivo as previously described above. For all analysis, the eosinophil stimulating factor interleukin-5 (IL-5) was measured in the patient plasma at baseline to avoid confounding effects. Criteria for hypereosinophilia and hypereosinophilic syndromes were applied as defined in the WHO Update 2019 (Shomali and Gotlib 2019), the diagnosis was confirmed by medical specialists.

Interestingly, the *MCL1* inhibitor S63845 specifically decreased the viability of bone marrow progenitor cells in patients with hypereosinophilia. In patients diagnosed with chronic eosinophilic leukemia (CEL-NOS) or myeloid and lymphatic neoplasia with hypereosinophilia (MLN-Eo) repression of survival was specifically powerful. Our study shows that *MCL1* inhibition might be a promising therapeutic option for hypereosinophilia patients specifically for CEL-NOS and MLN-Eo.

## Materials and methods

### Patient samples

Human peripheral blood (PB) and bone marrow (BM) samples were collected from patients with hypereosinophilia (Supplementary Table 1 and 2, HES *n* = 18, CEL-NOS *n* = 9, L-HES *n* = 2, MLN-Eo *n* = 2, EGPA^anca−^ *n* = 11, reactive eosinophilia *n* = 3) and healthy donors (*n* = 13) according to the institutional guidelines and in concordance with the Declaration of Helsinki. Healthy PB control samples were taken from volunteers at the TUM. Healthy BM control samples were collected from human femoral heads discarded after hip joint surgery. All subjects gave written informed consent. Clinical data of the subjects including treatment status can be found in the Supplementary (Supplementary Table 1). Patients on or after tyrosine kinase inhibition therapy have not been enrolled in this study.

### Cell culture

Granulocytes from PB and BMMCs were purified using Biocoll Separation Solution 1.077 g/ml (Biochrom, Berlin, Germany). Eosinophils were purified by negative selection using CD16 MicroBeads *(Miltenyi Biotec, Bergisch Gladbach, Germany). Granulocytes were cultured at a density of *5 × 10^5^ cells/ml media containing RPMI 1640 (Life Technologies, Carlsbad, USA), 20% FBS Good Forte (PAN Biotech, Aidenbach, Germany), 25 mM HEPES, 2 mM L-glutamine, 1 × MEM NEAA, 1 mM sodium pyruvate, 50 µM β-mercaptoethanol (all from Life Technologies, Carlsbad, USA) and 10 ng/ml rhIL-5 (Biolegend, San Diego, USA). BMMCs were kept at a density of 5 × 10^5^ cells/ml in media containing 20% BIT 9500 (StemCell, Vancouver, Canada), 100 ng/ml rhSCF, 5 ng/ml interleukin-6, 10 ng/ml interleukin-3, 10 ng/ml thrombopoietin (all from R&D Systems, Minneapolis, USA), 10 µM β-mercaptoethanol and 4 µg/ml low-density lipoproteins (StemCell, Vancouver, Canada).

### Inhibitors

ABT-199 (Abbvie, North Chicago, USA), ABT-737 (Active Biochem, Maplewood, USA), S63845 (Active Biochem, Maplewood, USA) and WEHI-539 (Aobious, Gloucester, USA) were dissolved in dimethyl sulfoxide (DMSO) and used in a final concentration of 1 µM. DMSO was used at 0.001% as soluble control.

### Cell viability assay

PB granulocytes were stained with anti-human Siglec-8 (APC, Clone 7C9; Miltenyi Biotec, Bergisch Gladbach, Germany), BMMCs were stained with CD34 (eFluor 450, Clone 4H11) and CD45 (APC, Clone 2D1; both: Invitrogen, Carlsbad, USA). Viability was measured by flow cytometry of Annexin V (FITC) and 7-aminoactinomycin D (7AAD; PerCP; both Invitrogen, Carlsbad, USA). Viable cells were negative for Annexin and 7AAD. Cytometry was performed on a FACS Canto II (BD Bioscience, Franklin Lakes, USA), data were analyzed using FlowJo Version 10.5.3 (FlowJo, Ashland, USA).

### Colony formation assay

After 72 h-treatment, 2 × 10^4^ BMMCs were seeded in MethoCult (H4435, StemCell, Vancouver, Canada) in duplicates. After 14 days, numbers of granulocyte–macrophage progenitor colonies (CFU-GM) were assessed manually by microscopy.

### RT-qPCR/ELISA

RNA from eosinophils was purified using an RNA isolation kit (Macherey–Nagel, Düren, Germany). RNA concentrations were normalized, cDNA was synthesized using the QuantiTect Reverse Transcription System (Qiagen, Venlo, Netherlands) in a Biometra TAdvanced Thermocycler (Analytic Jena, Jena, Germany). cDNA was analyzed with GoTaq qPCR Mastermix (Promega, Fitchburg, USA) using the in Supplementary Table 3 listed primers, *Morrbid* and *Bim* primers were analogous to Kotzin et al. (2016). Reactions were performed and analyzed using a LightCycler 480 Instrument II (Roche, Rotkreuz, Switzerland). The 2^−ΔΔCt^ method (Livak and Schmittgen 2001) was used for the relative quantification of the measured gene expression levels. To ensure comparability among the analysis, the housekeeping gene HPRT was included as internal control and the cell lines HL-60/KG-1 alpha were included as calibrator. Serum IL-5 was measured at baseline using a human IL-5 ELISA Kit (Biorbyt, Cambridge, UK). Reactions were performed and analyzed in a GloMax Discover Microplate Reader (Promega, Fitchburg, USA).

### Statistical analysis

The performed statistical tests are indicated in the figure legends. *p* values have not been adjusted for multiple testing, are two-sided and with a significance level of 0.05. Statistical analyses were performed using GraphPad Prism 7.0e (GraphPad, San Diego, USA).

## Results

As far as we know, no critical characterization is available comparing the central BCL-2 family members in healthy controls and patients with hypereosinophilia head-to-head. Here, we not only quantified RNA levels of BCL-2-family members in both groups, but also analyzed the effect of the BH_3_-mimetics ABT-199, ABT-737, WEHI-539 and S63845 on eosinophils of healthy donors and an enlarged cohort of patients with hypereosinophilic disorders (*n* = 45, Supplementary Tables 1 and 2).

### The role of Morrbid and BIM in aberrant eosinophils

**Fig. 1:**
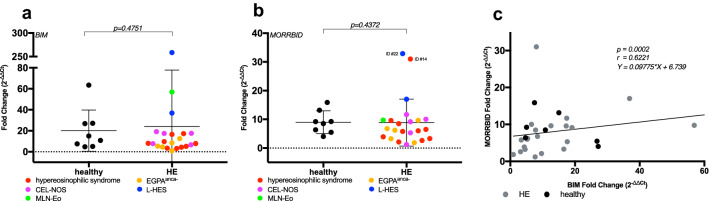
RNA expression analysis of BIM and Morrbid in primary human eosinophils. Gene expression of *BIM* and the long non-coding RNA *Morrbid* in primary human samples. Expression levels were measured by RT-qPCR in purified eosinophil granulocytes from healthy subjects and patients with hypereosinophilic disorders, all in duplicates. For analysis the 2^−∆∆Ct^ method was used, starting values with SD ≥ 0.5 between duplicates were excluded in the analysis due to quality reasons. In all analysis fold gene expression was normalized to the housekeeping gene *HPRT* and the HL-60 cell line. **a** BIM was measured in 8 healthy, age-matched controls and 22 patient samples (HE; HES *n* = 9; L-HES *n* = 2; MLN-Eo *n* = 1; CEL-NOS *n* = 5; EGPA^anca−^ *n* = 5) Mann–Whitney test was used, the *p* value is indicated. Data are shown with mean ± SD. **b** *Morrbid* was measured in 8 healthy, age-matched controls and 23 patient samples (HE; HES *n* = 10; L-HES *n* = 2; MLN-Eo *n* = 1; CEL-NOS *n* = 5; EGPA^anca−^ *n* = 5) Mann–Whitney test was used, the *p* value is indicated. Data are shown with mean ± SD. **c** For a correlation analysis of *BIM/Morrbid* in all eosinophils (healthy and aberrant), the strength of correlation was calculated by Pearson correlation (Pearson *r* = 0.6221) and the functional relation was described with linear regression (as indicated), the *p* value is *p* = 0.0002)

Since the long non-coding RNA *Morrbid* was recently described to determine eosinophil lifespan regulating the transcription of the pro-apoptotic gene *BIM* (*BCL2L11*) in an antagonistic manner, we analyzed both factors in healthy and aberrant eosinophils. Kotzin et al. (2016) hypothesized that hypereosinophilia in patients could be due to apoptotic resistance by upregulation of *Morrbid* and loss of *BIM.* Surprisingly, we did not identify any correlation between disease status and *BIM* or *Morrbid* levels (Fig. 1a, b). Only the two individual patients ID #22 (MLN-Eo) and patient ID #14 (HES) showed significantly elevated *Morrbid* levels. Similar findings for further individuals of the same disease subtype could not be reproduced. In addition, we identified a strong positive correlation between the long non-coding RNA *Morrbid* and *BIM* levels (Pearson *r* = 0.6221, *p* = 0.0002; Fig. 1c). The eosinophil stimulating factor IL-5 (measured at baseline) did not have any significant influence on *Morrbid* and *BIM* levels (Supplementary Fig. 1).

### Characterization of BCL-2-family proteins in eosinophil granulocytes using RT-qPCR

To investigate the role of further BCL-2 family proteins in eosinophils, we evaluated the expression of a panel of 9 central anti- and proapoptotic genes in purified eosinophil granulocytes by RT-qPCR (primer sequences see Supplementary Table 3).

**Fig. 2:**
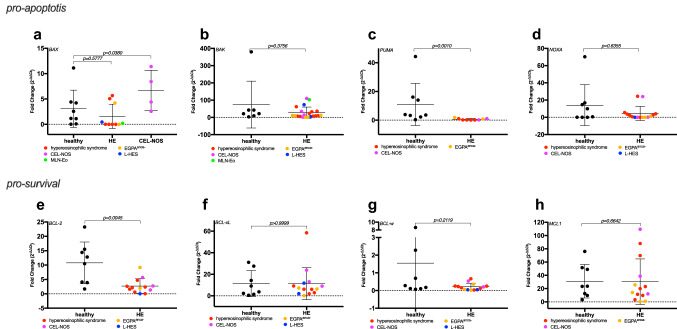
Gene expression of critical BCL-2 family members in primary human eosinophils. Gene expression of critical pro-apoptotic (**a**–**d**) and anti-apoptotic (**e**–**h**) BCL-2 family protein members in primary human samples. Expression levels were measured by RT-qPCR in purified eosinophil granulocytes from healthy subjects and patients with hypereosinophilic disorders, all in duplicates. For analysis the 2^−∆∆Ct^ method was used, starting values with SD ≥ 0.5 between duplicates were excluded in the analysis due to quality reasons. In all analysis fold gene expression was normalized to the housekeeping gene *HPRT* and the HL-60 cell line, data are shown with mean ± SD. **a** *BAX* was measured in 8 healthy, age-matched controls and 14 patient samples (HE; HES *n* = 6; L-HES *n* = 1; MLN-Eo *n* = 1; CEL-NOS *n* = 4; EGPA^anca−^ *n* = 2). Data were specially tested for differences between the healthy group and the patients with CEL-NOS. Kruskal–Wallis test and post-hoc pairwise comparison was applied, *p* values are indicated. **b** *BAK* was measured in 7 healthy, age-matched controls and 21 patient samples (HE; HES *n* = 9; L-HES *n* = 2; MLN-Eo *n* = 1; CEL-NOS *n* = 4; EGPA^anca−^ *n* = 5). Mann–Whitney test was used, the *p* value is indicated. **c** *PUMA* was measured in 8 healthy, age-matched controls and 9 patient samples (HE; HES *n* = 5; CEL-NOS *n* = 2; EGPA^anca−^ *n* = 2). Mann–Whitney test was used, the *p* value is indicated. **d** *NOXA* was measured in 8 healthy, age-matched controls and 15 patient samples (HE; HES *n* = 8; L-HES *n* = 1; CEL-NOS *n* = 3; EGPA^anca−^ *n* = 3). Here, analysis fold gene expression was normalized to the housekeeping gene *HPRT* and the KG1-alpha cell line. Mann–Whitney test was used, the *p* value is indicated. **e** *BCL-2* was measured in 8 healthy, age-matched controls and 13 patient samples (HE; HES *n* = 7; L-HES *n* = 1; CEL-NOS *n* = 3; EGPA^anca−^ *n* = 2). Mann–Whitney test was used, the *p* value is indicated. **f** *BCL-xL* was measured in 8 healthy, age-matched controls and 14 patient samples (HE; HES *n* = 6; L-HES *n* = 2; CEL-NOS *n* = 3; EGPA^anca−^ *n* = 3). Mann–Whitney test was used, the *p* value is indicated. **g** *BCL-w* was measured in 8 healthy, age-matched controls and 14 patient samples (HE; HES *n* = 7; L-HES *n* = 2; CEL-NOS *n* = 2; EGPA^anca−^ *n* = 3). Mann–Whitney test was used, the *p* value is indicated. **h** *MCL1* was measured in 8 healthy, age-matched controls and 14 patient samples (HE; HES *n* = 7; CEL-NOS *n* = 3; EGPA^anca−^ *n* = 4). Mann–Whitney test was used, the *p* value is indicated

First, we analyzed the pro-apoptotic effectors *BAX* and *BAK*, which are known to play a pivotal role in eosinophil apoptosis (Dewson et al. 1999, 2001). *BAX* and *BAK* mRNA levels were not different between healthy and aberrant eosinophils. However, higher *BAX* levels were detected in eosinophils from CEL-NOS patients (Fig. 2a, *p* = 0.0380). Both *BAX* and *BAK* are physiologically activated by BH_3_-only-proteins such as *BIM*, *PUMA* and *NOXA* and directly inhibited by pro-survival BCL-2 proteins (Llambi et al. 2011). While *BIM* and *NOXA* levels were not different between groups (Figs. 1a, 2b), we detected an almost complete loss of *PUMA* mRNA in the patient cohort (Fig. 2c, *p* = 0.0010). Taken together, these data suggest that aberrant eosinophils are unable to undergo *PUMA* induced apoptosis compared to normal eosinophils.

We then evaluated the pro-survival *BCL-2, BCL-xL, BCL-w* and *MCL1*. Whereas *BCL-xL*, *BCL-w* and *MCL1* showed similar expression levels between groups, *BCL-2* levels were significantly higher in healthy samples. Interestingly, we identified a common pattern in *BCL-2* and *BCL-w* expression levels. While they were widely scattered in healthy eosinophils, they were consistently low and clustered in the aberrant ones (Fig. 2d–g).

### Induction of apoptosis in eosinophil granulocytes of patient with hypereosinophilia using BH_3_ mimetics

Next, we investigated the effect of BCL-2 family protein inhibition on the eosinophil granulocytes’ survival. Eosinophils’ survival is highly dependent on interleukin-5 (IL-5) (Campbell et al. 1987). To prevent bias due to cytokine deprivation, we saturated the culture medium with 10 ng/ml IL-5 after stepwise titration. An influence of patient individual IL-5 blood plasma levels at the time point of specimen sampling on the later analysis could be excluded (Supplementary Fig. 2). Since eosinophil median survival in vivo is about 2 to 5 days but can last up to 14 days ex vivo (Park and Bochner 2010), an analysis time frame of 3 days was chosen after preliminary experiments. Patients’ samples were only included in the analysis when the absolute viability of the DMSO control was above 90% of all analyzed cells in order to normalize for effects of physiologic apoptosis. All BH_3_-mimetic compounds were titrated to a non-cytotoxic concentration in healthy BM and were applied at a concentration of 1 µM, in line with previously published studies (Jilg et al. 2019; Jilg et al. 2016; Rohner et al. 2020).

**Fig. 3:**
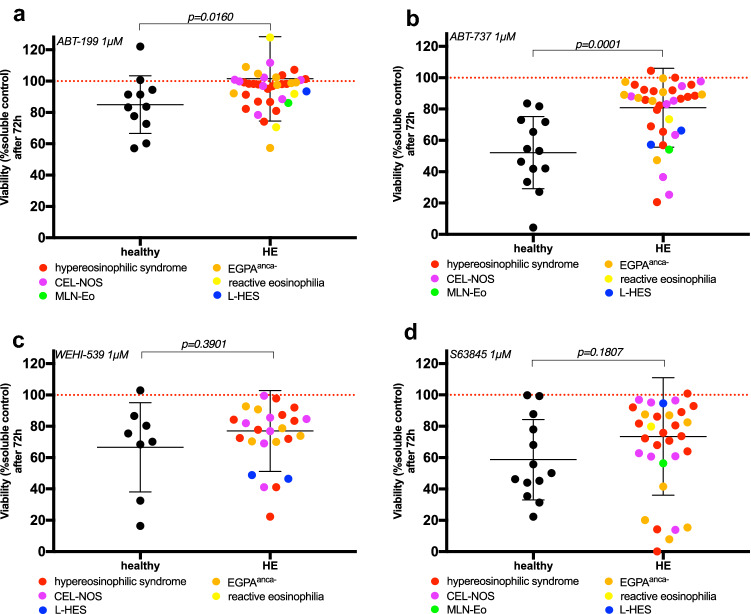
Aberrant eosinophilic granulocytes from patients with hypereosinophilic disorders are more resistant to apoptosis induction with BH_3_-mimetics compared to healthy controls. **a** Viable granulocytes of 11 healthy, age-matched controls and of 41 patients (HE; reactive eosinophilia *n* = 3, HES *n* = 18, L-HES *n* = 2, MLN-Eo *n* = 1, CEL-NOS *n* = 8, EGPA^anca−^ *n* = 9) were treated for 72 h with ABT-199 1 µM and DMSO soluble control and gated on Siglec8 + granulocytes. Cell viability was measured by flow cytometry using Annexin V and 7AAD staining, a ratio of inhibitory treatment to DMSO soluble control is shown with mean ± standard deviation (SD). Mann–Whitney test was used, the *p* value is indicated. **b** Viable granulocytes of 13 healthy, age-matched controls and of 38 patients (HE; reactive eosinophilia *n* = 1, HES *n* = 17, L-HES *n* = 2, MLN-Eo *n* = 1, CEL-NOS *n* = 8, EGPA^anca−^ *n* = 9) were treated for 72 h with ABT-737 1 µM and DMSO soluble control and gated on Siglec8 + granulocytes. Cell viability was measured by flow cytometry using Annexin V and 7AAD staining, a ratio of inhibitory treatment to DMSO soluble control is shown with mean ± standard deviation (SD). Mann–Whitney test was used for analysis and the *p* value is indicated. **c** Viable granulocytes of 8 healthy, age-matched controls and of 26 patients (HE; HES *n* = 10, L-HES *n* = 2, CEL-NOS *n* = 7, EGPA^anca−^ *n* = 7) were treated for 72 h with WEHI-539 1 µM and DMSO soluble control and gated on Siglec8 + granulocytes. Cell viability was measured by flow cytometry using Annexin V and 7AAD staining, a ratio of inhibitory treat to DMSO soluble control is shown with mean ± standard deviation (SD). Mann–Whitney test was used, the *p* value is indicated. **d** Viable granulocytes of 13 healthy, age-matched controls and of 35 patients (HE; reactive eosinophilia *n* = 1, HES *n* = 15, L-HES *n* = 2, MLN-Eo *n* = 1, CEL-NOS *n* = 7, EGPA^anca−^ *n* = 9) were treated for 72 h with S63845 1 µM and DMSO soluble control and gated on Siglec8 + granulocytes. Cell viability was measured by flow cytometry using Annexin V and 7AAD staining, a ratio of inhibitory treatment to DMSO soluble control is shown with mean ± standard deviation (SD). Mann–Whitney test was used, the *p* value is indicated

Notably, healthy eosinophils showed an overall susceptibility to the different inhibitors. Whilst inhibition of *BCL-2* with ABT-199 and inhibition of *BCL-xL* with WEHI 539 had only minor effects on cell viability, inhibition of *MCL1* with S63845 and pan-BCL-2 inhibition with ABT-737 strongly reduced cell viability with respect to DMSO treated control (red dashed line in Fig. 3a–d). In one healthy subject, ABT-737 administration even led to complete eosinophil eradication in vitro.

Interestingly, we identified a different pattern in patients with hypereosinophilic disorders. Across all treatment modalities, cell viability was overall higher in aberrant eosinophils. Cell viability was significantly increased by ABT-199 and ABT-737 treatments, under ABT-737 treatment especially patients diagnosed with EGPA did not respond to BCL-2 protein inhibition (Fig. 3a, b). Taken together these data suggested that survival of aberrant eosinophils is less dependent on *BCL-2* than in eosinophils from healthy donors. Both WEHI 539 and S63845 treatments did not elicit a significant difference between healthy and aberrant eosinophils (Fig. 3c, d). However, after *MCL1* inhibition, the eosinophils from patients displayed a bimodal distribution. While the majority of samples showed a significant increase in viability, a small subgroup showed a drastic decrease in cell numbers (Fig. 3e). We did not identify any correlation between the susceptibility to *MCL1* inhibition and diagnosis, clinical characteristics, pre-treatment or biomarkers (Supplementary Tables 1 and 2).

### The effect of BH_3_-mimetics on myeloid progenitor cells of patients with hypereosinophilia

**Fig. 4:**
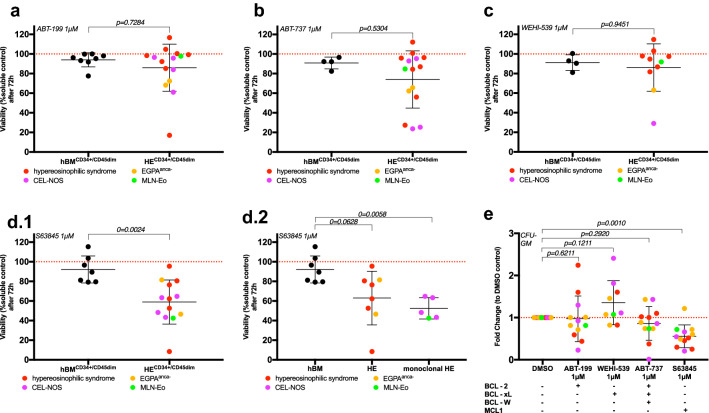
S63845 reduces cell viability in primary human myeloid progenitor cells. Healthy CD34^+^/CD45^dim^ progenitor cells remain unaffected by BCL-2 family protein inhibition whilst targeting MCL1 with S63845 reduces cell viability in patient samples. The MCL1 inhibitor S63845 shows the most long-term effect on BMMCs from patients with hypereosinophilic disorders. **a** Viable BMMCs of 8 healthy, age-matched controls (hBM) and of 15 patients (HE; HES *n* = 8, CEL-NOS *n* = 4, MLN-Eo *n* = 1 and EGPA^anca−^ *n* = 2) were treated 72 h with ABT-199 1 µM for 72 h and DMSO soluble control and gated on CD34^+^/CD45^dim^. Cell viability was measured by flow cytometry using Annexin V and 7AAD staining, a ratio of inhibitory treatment to DMSO soluble control is shown with mean ± standard deviation (SD). Mann–Whitney test was used, the *p* value is indicated. **b** Viable BMMCs of 4 healthy, age-matched controls and of 15 patients (HES *n* = 8, CEL-NOS *n* = 4, MLN-Eo *n* = 1 and EGPA^anca−^ *n* = 2) were treated with ABT-737 for 72 h and DMSO soluble control and gated on CD34^+^/CD45^dim^. Cell viability was measured by flow cytometry using Annexin V and 7AAD staining, a ratio of inhibitory treatment to DMSO soluble control is shown with mean ± standard deviation (SD). Mann–Whitney test was used, the *p* value is indicated. **c** Viable BMMCs of 4 healthy, age-matched controls and of 10 patients (HES *n* = 7, CEL-NOS *n* = 1, MLN-Eo *n* = 1 and EGPA^anca−^ *n* = 1) were treated with WEHI-539 for 72 h and DMSO soluble control and gated on CD34^+^/CD45^dim^. Cell viability was measured by flow cytometry using Annexin V and 7AAD staining, a ratio of inhibitory treatment to DMSO soluble control is shown with mean ± standard deviation (SD). Mann–Whitney test was used, the *p* value is indicated. **d** Viable BMMCs of 7 healthy, age-matched controls and of 13 patients (HES *n* = 6, CEL-NOS *n* = 4, MLN-Eo *n* = 1 and EGPA^anca−^ *n* = 2) were treated with S63845 for 72 h and DMSO soluble control and gated on CD34^+^/CD45^dim^. Cell viability was measured by flow cytometry using Annexin V and 7AAD staining, a ratio of inhibitory treatment to DMSO soluble control is shown with mean ± standard deviation (SD). For **d.1** Mann–Whitney test was used, for, **d.2** Kruskal–Wallis test and post-hoc pairwise comparison was performed. The *p* values are indicated. **e** BMMCs (2 × 10^4^) were plated in duplicates in growth-factor-enriched methylcellulose 72 h treatment with ABT-199 (*n* = 12), WEHI-539 (*n* = 9), ABT-737 (*n* = 12), S63845 (*n* = 12) in the indicated concentration and DMSO vehicle control. After 14 days, a total number of granulocyte–macrophage colony forming units (CFU-GM) was counted on every plate. The mean of every duplicate was calculated. A ratio of the colony count of inhibitory treatment to DMSO soluble control is shown with mean ± SD. Wilcoxon test was used, the *p* values are indicated

Whereas the pathogenesis of idiopathic hypereosinophilia and the hypereosinophilic syndrome remain widely unclear, in the case of CEL-NOS and MLN-Eo defects on the level of the myeloid progenitor cell are disease defining. Hence, we analyzed the effect of our panel of BH3 mimetics on myeloid progenitor cells. BMMCs from healthy donors and hypereosinophilic patients were kept in cytokine-enriched, serum-free medium as described previously (Dill et al. 2019; Jilg et al. 2019) and treated for 72 h. As expected, CD34^+^/CD45^dim^ myeloid hematopoietic progenitor cells from age-matched healthy donors were resistant to all the treatments (Fig. 4). Progenitor cells from patients showed a highly varying response to treatment with ABT-199 (mean 86.9%, SD 24.1%, Fig. 4a), ABT-737 (mean 74.0%, SD 29.2, Fig. 4b) and WEHI-539 (mean 86.1%, SD 24.2%; Fig. 4c). However, no overall significant effect was detected for either treatment. On the other hand, MCL1 inhibition with S63845 induced a significant reduction in cell viability (mean 58.9%, SD 22.5%; Fig. 4d.1), interestingly, this effect was mainly driven by the monoclonal variants of hypereosinophilia, such as CEL-NOS and MLN-Eo (mean 52.5%, SD 10.9%; Fig. 4d.2). To strengthen the possible translational value of our work, we analyzed the colony forming capacity as an index of long-term effects. In line with our previous data, the effect of ABT-199, ABT-737 and WEHI-539 is widely scattered, while *MCL1* inhibition showed a long-run inhibitory effect on the colony forming capacity (Fig. 4e). Taken together, these data implicate that *MCL1* inhibition selectively targets aberrant myeloid progenitor cells, while sparing the healthy hematopoiesis.

## Discussion

Hypereosinophilic disorders are a heterogenous group of disease with different pathogeneses and diverging involvement of the compartments peripheral blood, bone marrow and tissue. Therapy strategies are tripartite. (1) Often, the main therapeutic regimen is unspecific using general cytoreduction of hematopoietic cells and/or immunosuppression (e.g. hydroxycarbamide and corticosteroids). (2) Targeting the eosinophil survival factor IL-5 or the IL-5 receptor α within the scope of steroid-sparing eosinophil reduction is a promising approach (mepolizumab and benralizumab). These expensive medications are only approved under tight circumstances. (3) Only for a small patient group with strictly defined genetic aberrations (e.g. FIP1L1-PDGFRA/PDGFRB rearrangements), a real causal therapy is available with tyrosine kinase inhibitors (imatinib). Further possible therapeutical strategies (e.g. targeting the surface molecule Siglec 8) are in the focus of current research (Legrand and Klion 2015).

Programmed cell death is critical for a normal tissue homeostasis in multicellular organisms (Hotchkiss et al. 2009). A balance between cell death and cell proliferation is essential for an effective hematopoiesis and immune regulation, particularly for cells with short lifespan such as eosinophil granulocytes. Our work especially focuses on analyzing the apoptotic machinery. It is well targetable and smaller data sets suggest relevant impact of the *BCL-2* family members on eosinophil survival. Interestingly, *BCL-2* itself appears not to be expressed in eosinophils (Dibbert et al. 1998), although contrasting reports can be found (El-Gamal et al. 2004) as discussed by Park and Bochner (2010). In contrast, it is consistently shown, that healthy eosinophils express the anti-apoptotic proteins *BCL-xL* and *MCL1* and the pro-apoptotic *BAX* (Dibbert et al. 1998). In line with these findings, delayed eosinophil apoptosis as a cause for tissue eosinophilia was reported several years ago supporting the notion that aberrations to the apoptotic machinery contribute to pathogenicity (Simon et al. 1997).

In our study, we detected qualitative and quantitative differences between peripheral blood eosinophils from healthy subjects and from patients with hypereosinophilic disorders concerning their apoptotic behavior. A recent prominent report by Kotzin et al. (2016) reported that reductions in pro-apoptotic *BIM* control survival of aberrant eosinophils. Specifically, this publication stated that the long non-coding RNA *Morrbid* regulates the transcription of the pro-apoptotic gene *BCL2L11* (*BIM*) in an antidromic manner and that *Morrbid* is significantly upregulated in patients with hypereosinophilic disorders (Kotzin et al. 2016). This was considered to be the basis for subsequent evasion from apoptosis. Our data using a cohort of primary human blood or bone marrow samples from patients with hypereosinophilia failed to detect any difference in *Morrbid* or *BIM* levels compared to healthy subjects. Both cohorts are comparable in size and the heterogenous patient collective—in our experiments only two of the subjects show the previously expected *Morrbid* elevation. While we did not find differences in *Morrbid*/*BIM*, we detected a nearly complete loss of pro-apoptotic *PUMA* in the aberrant eosinophils. Similar to what has already been described before for other hematologic diseases (Guirguis et al. 2016), this finding is a first evidence that abnormalities in the PUMA- and/or p53 dependent apoptosis mechanism contribute to hypereosinophilia.

This opened the question as to whether further BCL-2 family proteins have critical impact on eosinophil survival. The data on survival of healthy eosinophils in response to BH_3_-mimetics is limited (Rohner et al. 2020) and, more importantly, the concept of pharmacologic induction of apoptosis as treatment option in hypereosinophilia remains largely undefined. Especially in monoclonal- and stem cell-driven hypereosinophilia such as CEL-NOS and MLN-Eo, this therapeutic concept might hold substantial therapeutic impact.

In our experiments aberrant peripheral blood eosinophils were more resistant to apoptosis induction than the healthy eosinophils suggesting that hypereosinophilia might be driven by apoptotic resistance in the periphery probably in connection with higher eosinophil proliferation rates in the BM. Of note, therapy response to all applied BH_3_-mimetics in vitro was completely independent of the patient’s pre-treatment, the clinical manifestation and the absolute eosinophil count at diagnosis and sampling (see patient data in Supplementary Table 1). We observed that BCL-2 inhibition by venetoclax failed to induce apoptosis in eosinophils from hypereosinophilic patients. This was in line with the reduced levels of *BCL-*2 present in these aberrant eosinophils. Interestingly, we identified a sub-cohort of patients highly sensitive to *MCL1* inhibition. In accordance with these results from the peripheral blood, the new *MCL1* inhibitor S63845 showed high short- and long-term efficacy on the malignant stem and progenitor cell compartment of patients with a hypereosinophilic disorder, especially in BMMCs from patients with monoclonal hypereosinophilia (CEL-NOS and MLN-Eo). Remarkably, BMMCs of a KIT D816V positive CEL-NOS patient in our group showed high response to *MCL1* inhibition. Here, the cell viability was significantly reduced to 65% compared to healthy bone marrow. Patients in our entire cohort have neither previously received TKI nor were they under current TKI treatment. Notably, *MCL1* treatment, in our settings, did not affect the viability of healthy CD34^+^ myeloid progenitor cells.

The loss of *PUMA* in eosinophil granulocytes and the beneficial response of the *MCL1* inhibitor S63845, particularly in BMMCs, showed that alterations in the apoptotic machinery exist and can be exploited therapeutically. Indeed, *MCL1* inhibition is currently in early clinical testing for multiple myeloma and MYC positive diffuse large B-cell lymphoma (DLBCL) with promising results (Clinicaltrails.gov, NCT02992483). Therefore, *MCL1* might represent a promising target for hypereosinophilic patients, specifically for the myeloproliferative forms. While further clinical trials will be needed to test the in vivo toxicity of *MCL1* inhibition and the impact on clinical reality, our work set the basis for a new approach for this disease.

## Supplementary Information

Below is the link to the electronic supplementary material.

Supplementary file1 (PDF 3501 kb)

## Data Availability

Further information regarding clinical characteristics of hypereosinophilia patients contributing samples are provided.
