# Supplementary material for: Comprehensive characterization of central BCL-2 family members in aberrant eosinophils and their impact on therapeutic strategies
Source: J Cancer Res Clin Oncol. 2021 Oct 15;148(2):331–40. doi: 10.1007/s00432-021-03827-9 (PMC8800915; doi:10.1007/s00432-021-03827-9)
Supplement: Supplementary file 1 — Supplementary file1 (PDF 3501 kb) [file 432_2021_3827_MOESM1_ESM.pdf]

Supplementary Information: Comprehensive characterization of central BCL-2 family members in aberrant eosinophils and their impact on therapeutic strategies

Journal of Cancer Research and Clinical Oncology

Timo O. Odinius[1,2], Lars Buschhorn[1,2], Celina Wagner[1,2], Richard T. Hauch[1], Veronika Dill[1,2], Marta Dechant[1,2], Michele C. Buck[1], Khalid Shoumariyeh[3,4], Philipp Moog[5], Juliana Schwab[6], Andreas Reiter[6], Knut Brockow[7], Katharina Götze[1], Florian Bassermann[1,2], Ulrike Höckendorf[1,2], Caterina Branca[1,2], Philipp J. Jost[1,2,8,*,#] and Stefanie Jilg [1,*,#]

[1]Clinic and Policlinic for Internal Medicine III, School of Medicine, Technical University of Munich, Munich, Germany.

[2]Centre for Translational Cancer Research (TranslaTUM), School of Medicine, Technical University of Munich, Munich, Germany.

[3]Department of Medicine I, Medical Center - University of Freiburg, Faculty of Medicine, University of Freiburg, Germany.

[4]German Cancer Consortium (DKTK) Partner Site Freiburg, Germany

[5]Department of Nephrology, Clinic and Policlinic for Internal Medicine II, School of Medicine, Technical University of Munich, Munich, Germany.

[6]Department of Hematology and Oncology, University Hospital Mannheim, Heidelberg University, Mannheim, Germany.

[7]Department of Dermatology and Allergy, School of Medicine, Technical University of Munich, Munich, Germany.

[8]Division of Clinical Oncology, Department of Medicine, Medical University of Graz, Austria.

[*] authors contributed equally

#Corresponding author:	Philipp Jost and Stefanie Jilg
Postal address:	Klinik und Poliklinik für Innere Medizin III<br>Hämatologie und Internistische Onkologie<br>Technische Universität München (TUM)<br>Ismaninger Straße 22, 81675 München, Germany
Email address:	philipp.jost@tum.de; stefanie.jilg@tum.de

Supplementary Information: Figures

Comprehensive characterization of central BCL-2 family members in aberrant eosinophils and their impact on therapeutic strategies

Timo O. Odinius, *et al*.

Supplementary Figure 1:

Interleukin-5 (IL-5) blood plasma levels at baseline did not influence the response to BH_3-mimetic treatment in 72h viability assays. Viability data from aberrant eosinophils (**see Figure 3**) were correlated by Pearson correlation with IL-5 in blood plasma levels at the timepoint of sampling. The functional relation was described with linear regression. **(A-D)** show the relation between IL-5 absorbance levels and viability after 72h treatment with ABT-199, ABT-737, S63845, WEHI-539, each at 1µM. Pearson r, p-values and linear regression terms are indicated.

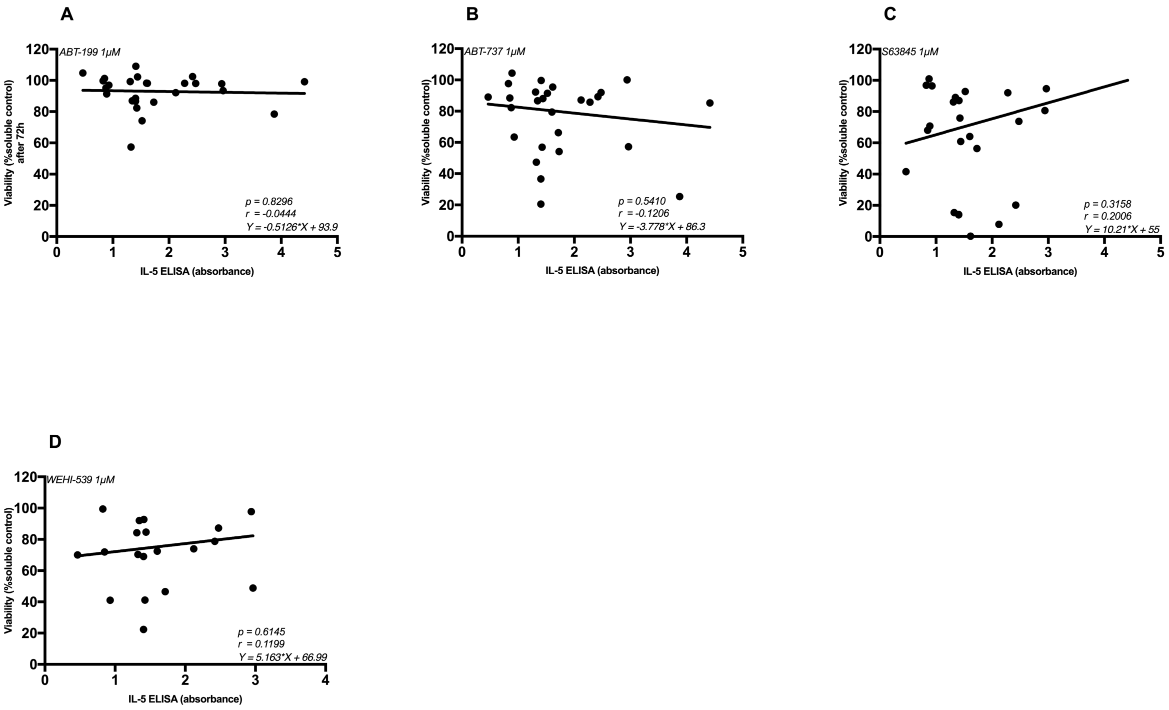

Supplementary Figure 2:

IL-5 blood plasma levels at baseline do not correlate with the expression level of BIM or *Morrbid*. **(A, B)** Gene expression data from aberrant and healthy eosinophils (**see Figure 1**) were correlated by Pearson correlation with IL-5 in blood plasma levels at the timepoint of sampling. The functional relation was described with linear regression. Pearson r, p-values and linear regression terms are indicated.

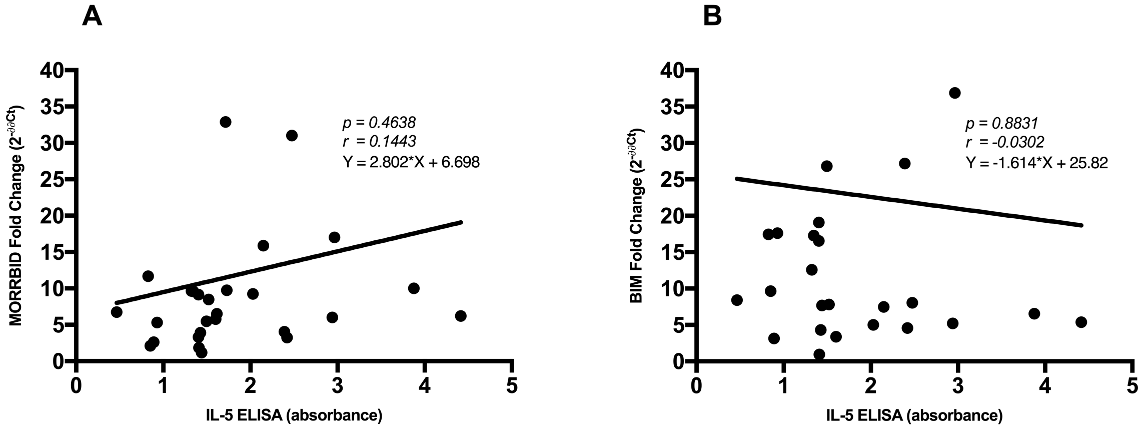

Supplementary Figure 3:

Supplement of **Figure 3** (main text): Aberrant eosinophilic granulocytes from patients with hypereosinophilic disorders are more resistant to apoptosis induction with BH3-mimetics than healthy eosinophils. **(A)** Viable granulocytes of 13 healthy, age-matched controls and of 38 patients (HE; reactive eosinophilia n=1, HES n=17, L-HES n=2, MLN-Eo n=1, CEL-NOS n=8, EGPA^{anca-} n=9) were treated for 72h with ABT-737 1µM and DMSO soluble control and gated on Siglec8+ granulocytes. Cell viability was measured by flow cytometry using Annexin V and 7AAD staining, a ratio of inhibitory treatment to DMSO soluble control is shown with mean ± standard deviation (SD). Data were especially tested for differences between the healthy and the EGPA^{anca-} patient group. Kruskal-Wallis test and post-hoc pairwise comparison was applied, the p-values are indicated. **(B)** Viable granulocytes of 13 healthy, age-matched controls and of 35 patients (HE; reactive eosinophilia n=1, HES n=15, L-HES n=2, MLN-Eo n=1, CEL-NOS n=7, EGPA^{anca-} n=9) were treated for 72h with S63845 1µM and DMSO soluble control and gated on Siglec8+ granulocytes. Cell viability was measured by flow cytometry using Annexin V and 7AAD staining, a ratio of inhibitory treatment to DMSO soluble control is shown with mean ± standard deviation (SD). The analysed cohort was subdivided according to the result of the 72h viability assay. Good responders were classified when viability was < 60%, bad responders when viability was >60%. Kruskal-Wallis test and post-hoc pairwise comparison was used, p-values are indicated.

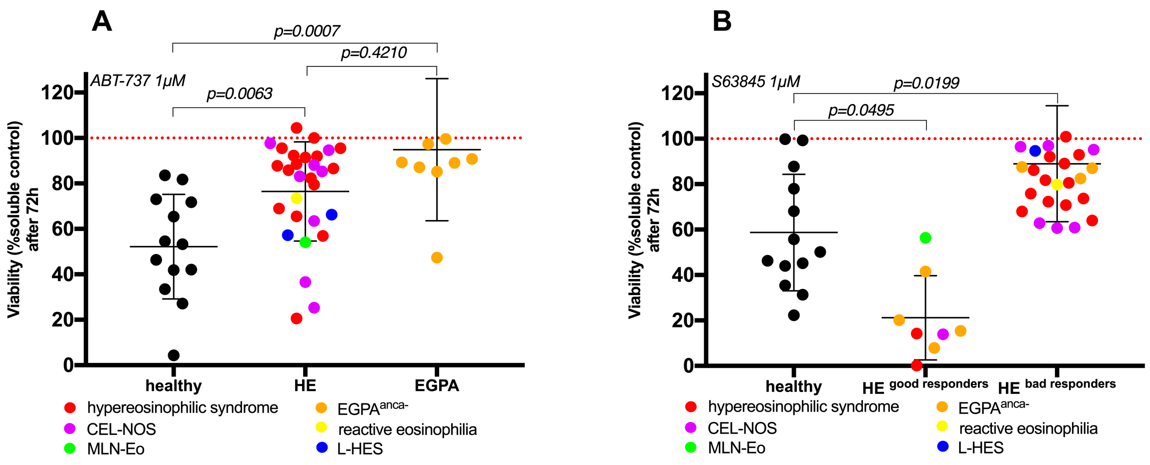

Supplementary Figure 4:

Shown is an example of the FACS analysis gating strategy for viability analysis. After excluding cell debris (1 and 2), Siglec-8[+] eosinophils (3) are gated. Cell death was measured by flow cytometry of Annexin V (FITC) and 7-aminoactinomycin D (7AAD; PerCP). Viable cells were negative for both Annexin and 7AAD (4, sector Q4).

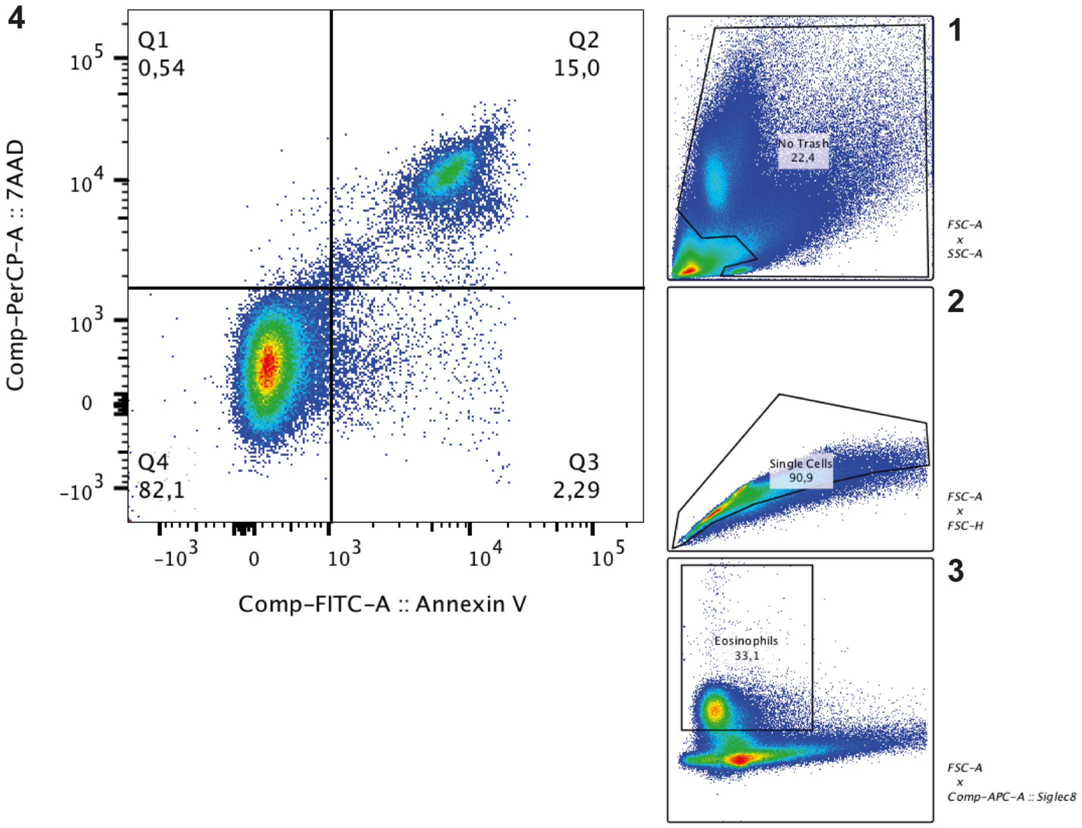

Supplementary Information: Tables

Comprehensive characterization of central BCL-2 family members in aberrant eosinophils and their impact on therapeutic strategies

Timo O. Odinius, *et al*.

Supplementary Table 1:

Clinical and molecular characteristics of hypereosinophilic patients contributing samples. Age, sex, WHO category, maximal eosinophil count (in % of the total leucocyte count and absolute eosinophil count), location of clinical manifestation and additional information were documented for each patient sample. Criteria for hypereosinophilia and hypereosinophilic syndromes were applied as defined in the WHO Update 2019. m = male, f = female, HES = hypereosinophilic syndrome, L-HES = lymphocyte variant hypereosinophilic syndrome, MLN-Eo = eosinophilia-associated myeloproliferative neoplasia, CEL-NOS = chronic eosinophilic leukemia not otherwise specified, EGPA = eosinophilic granulomatosis with polyangiitis, Eos = Eosinophil granulocytes, BMMCs = Bone marrow mononuclear cells, R-CHOP = rituximab/cyclophosphamide/hydroxydaunorubicin/ vincristine/prednisone, NA= not available, GI = gastrointestinal tract, PNS = peripheral nervous system, ENT = ear, nose, throat, BM = bone marrow, AKI = acute kidney injury, DLBCL = diffuse large B-cell lymphoma, ANCA = anti-neutrophil cytoplasmatic antibody.

ID	Age	Sex	Type of Hypereosinophilia	Max. (%) Eosinophils in Blood	Max. Eosinophils in Blood (G/L)	IL-5 level (absorbance)	Viability Eos (%DMSO) after 72h ABT-199	Viability Eos (%DMSO) after 72h ABT-737	Viability Eos (%DMSO) after 72h WEHI-539	Viability Eos (%DMSO) after 72h S63845	Viability BMMCs (%DMSO) after 72h ABT-199	Viability BMMCs (%DMSO) after 72h ABT-737	Viability BMMCs (%DMSO) after 72h WEHI-539	Viability BMMCs (%DMSO) after 72h S63845	(Pre-)Treatment	Location of Main Symptoms / Eosinophil infiltration	Comment
1	66	m	reactive	72	3,9	NA	70,59	NA	NA	NA	NA	NA	NA	NA	corticosteroids	GI	Primary sclerosing cholangitis
2	60	f	reactive	25	2,5	NA	91,81	NA	NA	NA	NA	NA	NA	NA	corticosteroids	GI, skin	
3	27	f	reactive	NA	NA	NA	127,87	73,44	NA	79,84	NA	NA	NA	NA	NA	NA	Hodgkin lymphoma
4	67	m	HES	NA	NA	0,88	95,10	82,28	NA	100,93	NA	NA	NA	NA	NA	lung	
5	33	f	HES	47	15,0	2,28	98,20	85,79	NA	92,06	NA	NA	NA	NA	corticosteroids	GI, skin	
6	49	m	HES	24	2,2	NA	103,84	NA	NA	NA	NA	NA	NA	NA	corticosteroids	skin, PNS	Angioedema
7	58	f	HES	15	0,8	NA	107,20	95,53	77,81	81,70	17,06	27,38	NA	8,41	none	ENT, lung, kidney	Mast cell proliferation in BM, but no mastocytosis
8	28	f	HES	9	0,3	NA	96,82	87,79	NA	NA	NA	NA	NA	NA	fexofenadine	skin, lung, recurrent anaphylaxis	Mast cell proliferation in BM, but no mastocytosis
9	76	m	HES	8	0,5	1,52	74,13	91,44	NA	92,90	NA	NA	NA	NA	none	allergy, skin, lung	Angioedema
10	36	m	HES	38	5,7	0,89	91,36	104,39	NA	70,74	98,40	95,83	NA	76,47	NA	heart	
11	33	m	HES	39	5,7	2,94	97,90	100,00	97,79	80,62	NA	NA	NA	NA	NA	GI, lung, kidney	AKI
12	49	m	HES	29	1,8	0,85	101,29	88,49	71,93	68,05	104,72	112,18	114,73	NA	corticosteroids	kidney	Thrombopenia
13	62	f	HES	NA	NA	1,43	82,34	56,93	41,17	75,82	NA	NA	NA	NA	NA	heart	
14	62	m	HES	67	15,8	2,48	98,07	91,93	87,23	73,73	NA	NA	NA	NA	none	allergy, GI, lung	
15	57	f	HES	35	2,6	1,60	98,29	79,46	72,49	64,06	92,34	56,09	103,01	80,57	none	allergy, heart, ENT, lung	
16	62	m	HES	49	6,2	1,35	86,96	86,61	92,00	89,04	NA	NA	NA	NA	corticosteroids, mesalazine, adalimumab	GI, lung, PNS	Primary sclerosing cholangitis
17	35	m	HES	NA	NA	1,41	86,67	20,54	22,35	14,20	99,29	96,33	94,79	62,25	corticosteroids	skin	
18	35	m	HES	NA	NA	NA	161,16	69,01	83,47	72,31	116,70	86,99	86,80	52,62	none	GI	
19	68	f	HES	NA	NA	NA	81,01	65,50	NA	NA	85,20	84,48	NA	NA	none	NA	Multiple myeloma
20	47	m	HES	8	0,4	1,31	99,20	92,24	84,25	86,19	NA	NA	NA	NA	NA	allergy, skin, lung	
21	54	m	HES	35	2,6	1,62	98,15	95,49	NA	0,21	100,34	100,79	NA	95,48	none	heart	
22	40	f	L-HES	NA	NA	1,72	186,05	66,28	46,51	150,00	NA	NA	NA	NA	none	NA	
23	69	f	L-HES	18	1,9	2,97	93,50	57,23	48,84	94,65	NA	NA	NA	NA	R-CHOP	allergy	DLBCL
24	78	f	MLN-Eo	60	8,0	1,73	86,07	54,11	NA	56,39	NA	NA	NA	NA	corticosteroids	GI	FIP1L1-PDGFRA +
25	56	m	MLN-Eo	NA	NA	NA	NA	NA	NA	NA	97,78	84,71	91,86	42,54	NA	NA	Anaemia, thrombopenia, splenomegaly; FILP1-PDGFRA +
26	57	m	CEL-NOS	34	4,5	NA	NA	NA	NA	NA	NA	NA	NA	NA	corticosteroids	heart	DNMT3A mutation
27	70	f	CEL-NOS	90	68,0	3,88	78,41	25,31	NA	NA	NA	NA	NA	NA	hydroxyurea	NA	>5% blasts in BM

28	80	m	CEL-NOS	28	7,4	1,44	102,24	88,04	84,68	60,90	NA	NA	NA	NA	corticosteroids	NA	Anaemia, splenomegaly; ASXL-1, CBL, SRSF2, TET2, KIT D816V mutations
29	62	m	CEL-NOS	NA	NA	NA	111,75	94,61	85,56	60,71	NA	NA	NA	NA	NA	NA	JAK2 p.F556V exon 13 mutation
30	67	m	CEL-NOS	27	2,5	0,83	99,78	97,67	99,45	96,89	96,58	95,29	97,43	63,42	corticosteroids	allergy lung	5-10% blasts in BM
31	62	f	CEL-NOS	NA	NA	NA	100,68	83,13	76,87	62,86	95,77	92,96	97,89	64,79	none	allergy, heart	TET2, KIT D816V mutations
32	82	m	CEL-NOS	40	10,0	1,41	88,51	36,66	69,09	13,92	83,97	23,71	81,80	43,41	none	NA	34% blasts in BM
33	62	m	CEL-NOS	6	0,6	0,93	96,93	63,45	41,08	96,49	NA	NA	NA	NA	corticosteroids	lung	DNMT3A, SF3B1 mutations
34	75	f	CEL-NOS	80	32	NA	100,88	85,29	81,97	95,24	60,96	25,31	29,06	48,31	none	NA	JAK2-V617F mutation
35	65	f	EGPA	36	3,9	2,42	102,41	89,26	78,77	20,14	NA	NA	NA	NA	rituximab	ENT, lung, PNS	ANCA negative
36	49	f	EGPA	17	1,7	1,86	207,09	167,72	161,42	170,08	NA	NA	NA	NA	IVIG	ENT, lung	ANCA negative
37	82	f	EGPA	7	0,5	NA	NA	NA	NA	NA	NA	NA	NA	NA	mepolizumab	ENT, lung	ANCA negative
38	48	f	EGPA	28	1,5	NA	NA	NA	NA	NA	NA	NA	NA	NA	none	ENT, lung	ANCA negative
39	55	f	EGPA	16	1,4	NA	97,52	97,38	NA	87,60	NA	NA	NA	NA	corticosteroids	ENT, lung	ANCA negative
40	43	f	EGPA	52	11,5	4,42	99,13	85,22	NA	130,00	68,22	62,29	NA	46,61	azathioprin	allergy, ENT, heart, lung	ANCA negative
41	47	m	EGPA	8	0,4	1,41	109,06	99,61	92,76	87,06	NA	NA	NA	NA	none	ENT, lung	ANCA negative
42	66	f	EGPA	NA	NA	1,33	57,36	47,36	70,38	15,38	NA	NA	NA	NA	mepolizumab	skin, heart, ENT, lung, kidney	ANCA negative
43	78	f	EGPA	24	3,1	0,47	104,76	89,09	70,07	41,54	NA	NA	NA	NA	corticosteroids	heart, lung	ANCA negative
44	77	m	EGPA	35	6,0	2,12	92,16	87,16	73,92	7,85	NA	NA	NA	NA	corticosteroids	skin, lung, kidney, PNS	ANCA negative
45	57	f	EGPA	66	19,8	NA	98,74	90,77	90,77	82,55	72,43	65,65	63,08	81,54	none	skin, lung	ANCA negative

Supplementary Table 2:

Gene expression of critical BCL-2 family members in primary human eosinophils of hypereosinophilic patients contributing samples. Age, sex, WHO category and gene expression levels were documented for each patient sample. Gene expression levels are expressed as fold change. Criteria for hypereosinophilia and hypereosinophilic syndromes were applied as defined in the WHO Update 2019. m = male, f = female, HES = hypereosinophilic syndrome, L-HES = lymphocyte variant hypereosinophilic syndrome, MLN-Eo = eosinophilia-associated myeloproliferative neoplasia, CEL-NOS = chronic eosinophilic leukemia not otherwise specified, EGPA = eosinophilic granulomatosis with polyangiitis, NA = not available.

ID	Age	Sex	Type of Hypereosinophilia	BCL-2	BCL-xL	MCL1	BIM	MORRBID	BCL-w	PUMA	NOXA	BAK	BAX
1	66	m	reactive	NA	NA	NA	NA	NA	NA	NA	NA	NA	NA
2	60	f	reactive	NA	NA	NA	NA	NA	NA	NA	NA	NA	NA
3	27	f	reactive	NA	NA	NA	NA	NA	NA	NA	NA	NA	NA
4	67	m	HES	NA	NA	NA	NA	NA	NA	NA	NA	NA	NA
5	33	f	HES	NA	NA	NA	NA	NA	NA	NA	NA	NA	NA
6	49	m	HES	NA	NA	NA	NA	NA	NA	NA	NA	NA	NA
7	58	f	HES	NA	NA	NA	NA	NA	NA	NA	NA	NA	NA
8	28	f	HES	NA	NA	NA	NA	NA	NA	NA	NA	NA	NA
9	76	m	HES	NA	NA	NA	7,808	8,486	0,226	0,115	0,023	36,631	0,003
10	36	m	HES	1,602	6,042	9,781	3,138	2,621	0,250	NA	0,646	10,267	5,063
11	33	m	HES	2,362	9,158	19,973	5,205	6,000	0,157	0,074	2,071	7,362	0,006
12	49	m	HES	NA	NA	3,227	9,646	2,129	NA	NA	4,000	NA	5,676
13	62	f	HES	4,724	6,169	23,103	4,317	3,904	0,255	0,162	2,657	7,111	NA
14	62	m	HES	0,624	58,485	69,792	8,056	31,017	0,664	1,025	NA	62,250	NA
15	57	f	HES	2,612	2,898	11,432	3,387	5,796	0,032	0,167	3,706	5,676	0,019
16	62	m	HES	0,032	NA	NA	17,268	9,580	NA	NA	24,420	23,183	NA
17	35	m	HES	2,107	1,905	88,035	16,564	3,317	0,120	NA	5,502	9,580	0,011
18	35	m	HES	NA	NA	NA	NA	NA	NA	NA	NA	NA	NA
19	68	f	HES	NA	NA	NA	NA	NA	NA	NA	NA	NA	NA
20	47	m	HES	NA	NA	NA	NA	NA	NA	NA	NA	NA	NA
21	54	m	HES	NA	NA	NA	NA	6,521	NA	NA	NA	33,014	NA
22	40	f	L-HES	NA	2,035	NA	258,676	32,900	0,050	NA	NA	73,772	NA
23	69	f	L-HES	0,022	11,794	NA	36,886	17,030	0,046	NA	0,039	4,485	0,460
24	78	f	MLN-Eo	NA	NA	NA	56,886	9,747	NA	NA	NA	102,893	0,206
25	56	m	MLN-Eo	NA	NA	NA	NA	NA	NA	NA	NA	NA	NA
26	57	m	CEL-NOS	NA	NA	NA	NA	NA	NA	NA	NA	NA	NA
27	70	f	CEL-NOS	NA	NA	NA	6,566	10,021	NA	NA	0,232	NA	4,408
28	80	m	CEL-NOS	NA	NA	NA	7,674	1,197	NA	NA	NA	110,278	NA
29	62	m	CEL-NOS	NA	NA	NA	NA	NA	NA	NA	NA	NA	NA
30	67	m	CEL-NOS	5,676	13,132	38,720	17,448	11,672	NA	0,172	24,001	14,320	8,427
31	62	f	CEL-NOS	NA	NA	NA	NA	NA	NA	NA	NA	NA	NA
32	82	m	CEL-NOS	2,732	23,835	109,516	19,093	9,158	0,536	1,068	NA	35,261	11,392
33	62	m	CEL-NOS	1,329	9,000	12,084	17,630	5,315	0,209	NA	0,055	2,049	2,576
34	75	f	CEL-NOS	NA	NA	NA	NA	NA	NA	NA	NA	NA	NA
35	65	f	EGPA	NA	0,241	1,003	4,579	3,238	NA	NA	0,018	11,592	0,001
36	49	f	EGPA	NA	NA	NA	NA	NA	NA	NA	NA	NA	NA
37	82	f	EGPA	NA	NA	NA	NA	NA	NA	NA	NA	NA	NA
38	48	f	EGPA	NA	NA	NA	NA	NA	NA	NA	NA	NA	NA
39	55	f	EGPA	NA	NA	NA	NA	NA	NA	NA	NA	NA	NA
40	43	f	EGPA	9,190	6,364	25,546	5,389	6,212	0,361	0,776	1,197	9,747	4,213
41	47	m	EGPA	NA	NA	NA	0,946	1,840	0,019	NA	NA	0,993	NA
42	66	f	EGPA	1,952	7,285	14,123	12,597	9,646	0,062	1,873	0,030	11,794	NA
43	78	f	EGPA	NA	NA	0,455	8,427	6,751	NA	NA	NA	1,083	NA
44	77	m	EGPA	NA	NA	NA	NA	NA	NA	NA	NA	NA	NA
45	57	f	EGPA	NA	NA	NA	NA	NA	NA	NA	NA	NA	NA

Supplementary Table 3:

Following primer sequences were used for RT-qPCR analysis as described in **Material and Methods.**

**Target RNA**	**Primer sequence**
HPRT	5’ - GCT ATA AAT TCT TTG CTG ACC TGC TG - 3’
	5’ - AAT TAC TTT TAT GTC CCC TGT TGA CTG G - 3’
BCL2L11 (BIM)	5’ - GGT CCT CCA GTG GGT ATT TCT CTT - 3’
	5’ - ACT GAG ATA GTG GTT GAA GGC CTG G - 3’
BCL-2	5’ - GAT AAC GGA GGC TGG GAT G - 3’
	5’ - TCA CTT GTG GCC CAG ATA GG - 3’
MCL1	5’ - AGA AAG CTG CAT CGA ACC AT - 3’
	5’ CCA GCT CCT ACT CCA GCA AC - 3’
BCL-xL	5’ - CTT GGA TGG CCA CTT ACC TG - 3’
	5’ - AAG AGT GAG CCC AGC AGA AC - 3’
BCL-w	5’ - GGA CAA GTG CAG GAG TGG AT - 3’
	5’ - GTC CTC ACT GAT GCC CAG TT - 3’
BAK	5’ - ACC AGC CTG TTT GAG AGT GG - 3’
	5’ - GGC CTA GGA AGC CAG TCA G - 3’
BAX	5’ - TTT GCT TCA GGG TTT CAT CC - 3’
	5’ - ATC CTC TGC AGC TCC ATG TT - 3’
NOXA	5’ - AAG AAG GCG CGC AAG AAC - 3’
	5’ - TCC TGA GCA GAA GAG TTT GGA - 3’
PUMA	5’ - GGG GAG GAG GAA CAG TGG - 3’
	5’ - AGG AGT CCC ATG ATG AGA TTG T - 3’
MORRBID	5’ - ACT GGA TGG TCG CTG CTT TT - 3’
	5’ - CTT CCC AGG AAC TGT GCT GT - 3’
